# Mycotic aneurysm secondary to melioidosis in China: A series of eight cases and a review of literature

**DOI:** 10.1371/journal.pntd.0008525

**Published:** 2020-08-12

**Authors:** Hua Wu, Xuming Wang, Xiaojun Zhou, Zhicheng Wu, Yanyan Wang, Mengjie Pan, Binghuai Lu

**Affiliations:** 1 Department of Laboratory Medicine, Hainan General Hospital, Affiliated Hainan Hospital of Hainan Medical College, Haikou, China; 2 Department of Laboratory Medicine, First Affiliated Hospital of Hainan Medical College, Haikou, China; 3 Department of Pathology, Hainan General Hospital, Affiliated Hainan Hospital of Hainan Medical College, Haikou, China; 4 Department of Radiology, Hainan General Hospital, Affiliated Hainan Hospital of Hainan Medical College, Haikou, China; 5 Laboratory of Clinical Microbiology and Infectious Diseases, Department of Pulmonary and Critical Care Medicine, China-Japan Friendship Hospital; National Clinical Research Center of Respiratory Diseases, Beijing, China; University of Oxford, UNITED KINGDOM

## Abstract

*Burkholderia pseudomallei* is the causative agent of melioidosis, endemic in Southeast Asia and Northern Australia, and increasingly recognized in southern China, especially in Hainan Province. Mycotic aneurysm caused by *B*. *pseudomallei* is a rare but potentially severe illness with a high mortality rate. The clinical features of the mycotic aneurysm secondary to melioidosis have not been illustrated in China. Over a seven-year period (2013 to 2019), 159 patients with bacteremic melioidosis were retrospectively analyzed in Hainan province, China, of whom eight patients were confirmed to have mycotic aneurysm through the combination of imaging examination, pathologic examination and aneurysm tissue culture. We summarized these eight patients’ clinical characteristics, demographical features, treatments and outcomes. The susceptibilities to five commonly-used antibiotics for these eight *B*. *pseudomallei* isolates were also determined by E-test strips. Furthermore, the mycotic aneurysm cases secondary to melioidosis retrieved from the literature were also reviewed. Of the eight cases, six had abdominal mycotic aneurysms, one had a left iliac aneurysm, and the other one had an infectious mesenteric aneurysm. They were aged from 48 to 69 years old, and had the underlying risk factors of diabetes mellitus (2 patients), long-term smoking (4 patients), hypertension (6 patients), and soil and water contact history (6 patients), respectively. The positive arterial aneurysm imaging was observed in all patients *via* computed tomography (CT) or angiography. Eight *B*. *pseudomallei* isolates collected from both blood and mycotic aneurysm tissues remained 100% susceptible to imipenem and ceftazidime. After surgery combined with antibiotic administration, six patients survived, with a mortality rate of 25%. In melioidosis endemic areas, the mycotic aneurysm secondary to melioidosis might be underdiagnosed, and increased awareness of predisposing risk factors and clinical features of the mycotic aneurysm is required. Following a positive *B*. *pseudomallei* blood culture, the diagnosis of mycotic aneurysm should be under consideration in those with abdominal pain and/or hypertension. Imaging by CT or angiography is indispensable for its timely diagnosis and management.

## Author summary

*Burkholderia pseudomallei* has the potential to cause mycotic aneurysm, an infrequently-occurred complication of melioidosis, with a high mortality rate despite appropriate antibiotic therapy and vascular surgery. The comprehensive clinical features of mycotic aneurysm due to *B*. *pseudomallaei* have not been documented in the People’s Republic of China and might be underrecognized. Therefore, we described eight patients with mycotic aneurysm from 159 bacteremic melioidosis patients in Hainan, China, over a seven-year period (between 2013 to 2019). We summarized their clinical characteristics, demographical features, treatments and outcomes. To our knowledge, this is the first comprehensive report of mycotic aneurysm secondary to melioidosis in China. In summary, in melioidosis endemic areas, or for those returning from endemic areas, increased awareness of the risk factors is required for early diagnosis and management of mycotic aneurysm secondary to melioidosis.

## Introduction

*Burkholderia pseudomallei* is an aerobic Gram-negative, invasive bacillus, found in soil and stagnant water in tropical regions, and predominantly endemic to Southeast Asia and Australia [1–4]. Melioidosis, transmitted *via* percutaneous inoculation and inhalation of soil or water containing *B*. *pseudomallei* in the environment, is a potentially fatal infection, manifesting as acute, subacute, or chronic disease [5]. Those in regular contact with contaminated soil and water are most commonly affected [2]. Mycotic aneurysm, mainly aortic aneurysm, is an irreversible dilatation of an artery and a rare complication, of which the most frequent pathogens are *Salmonella* species and *Staphylococcus aureus*; it can also be caused by *B*. *pseudomallei* [4, 6, 7]. It was documented initially in 1995 that *B*. *pseudomallei* (named *Pseudomonas pseudomallei* at that time) caused pseudoaneurysm of the renal artery [8]. Afterwards, similar cases were reported worldwide [1, 9–11]. Most infected mycotic aneurysms by *B*. *pseudomallei* are located in the abdominal aorta [6, 12]. Nowadays, it is estimated that approximately 0.4%~7.5% melioidosis patients have a mycotic aneurysm [11, 13].

Mycotic aneurysm remains a difficult disease to manage. In a patient with bacteremic melioidosis and the presence of an aneurysm, a potential mycotic aneurysm should be under consideration. The mortality rates of melioidosis, mycotic aneurysm due to melioidosis, and mycotic aneurysm due to other pathogens were 10% to more than 40% [14], 23.5% and 18.2%, respectively [12]. No statistical difference was observed between *B*. *pseudomallei* and other pathogens [12]. In the People’s Republic of China, the mycotic aneurysm cases were rarely documented [3, 15]. However, this is an underestimated indigenous disease and should raise clinical concerns. Herein, we report eight blood and aneurysm tissue culture positive *B*. *pseudomallei* cases with mycotic aneurysm, and summarize their epidemiological and clinical manifestations as well as their treatments and outcomes. To the best of our knowledge, this is the first comprehensive evaluation of mycotic aneurysm due to *B*. *pseudomallei* in China.

## Methods and materials

### Ethical approval

The institutional review boards at the Hainan General Hospital approved the human subjects study protocol. Written informed consent was obtained from the patients or their direct relatives for publication of this study. A copy of the written consent is available for review by the editor of this journal.

### Case definition

Mycotic aneurysm is an aneurysm arising from infection of the arterial wall, usually bacterial. It is diagnosed when an aneurysm is present in the context of inflammation and positive blood cultures. Furthermore, melioidosis might be associated with pseudoaneurysm formation as a manifestation of disease in a minority of patients, in addition to infecting existing aneurysms or arterial plaques. The term "mycotic aneurysm" is used throughout for consistency in the current study.

### Epidemiological and clinical data

During the period from January 2013 to March 2019, we collected 159 patients visiting hospitals in Hainan province, China, with culture-confirmed bacteremic melioidosis, of whom eight patients had mycotic aneurysms.

**Fig 1:**
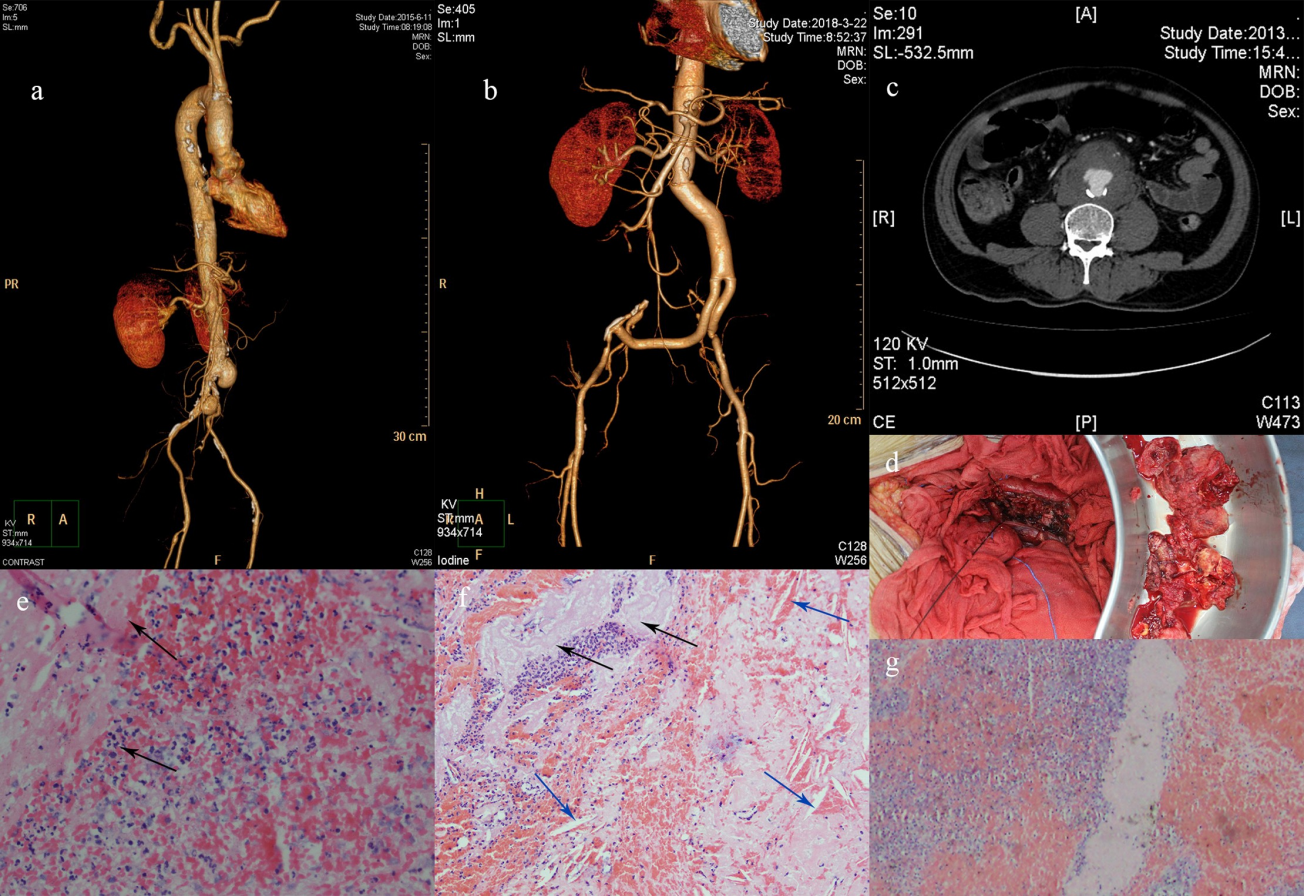
Angiography and CT scanning, aneurysm tissue collected in operation and pathological findings. Fig 1A and 1B: Mycotic aneurysm angiography before (left) and after (right) surgical management in patient 3; Fig 1C: CT scan revealed abdominal mycotic aneurysm with abdominal wall thrombosis in Patient 5; Fig 1D: Mycotic aneurysm tissue resected in Patient 5; Fig 1E–1G: Pathology of aneurysm tissues in Patient 5. 1e: hemorrhage and inflammatory cell infiltration; 1f: cholesterol crystal (blue arrow) and calcification (black arrow); 1g: collagenization of aneurysm tissues.

**Fig 2:**
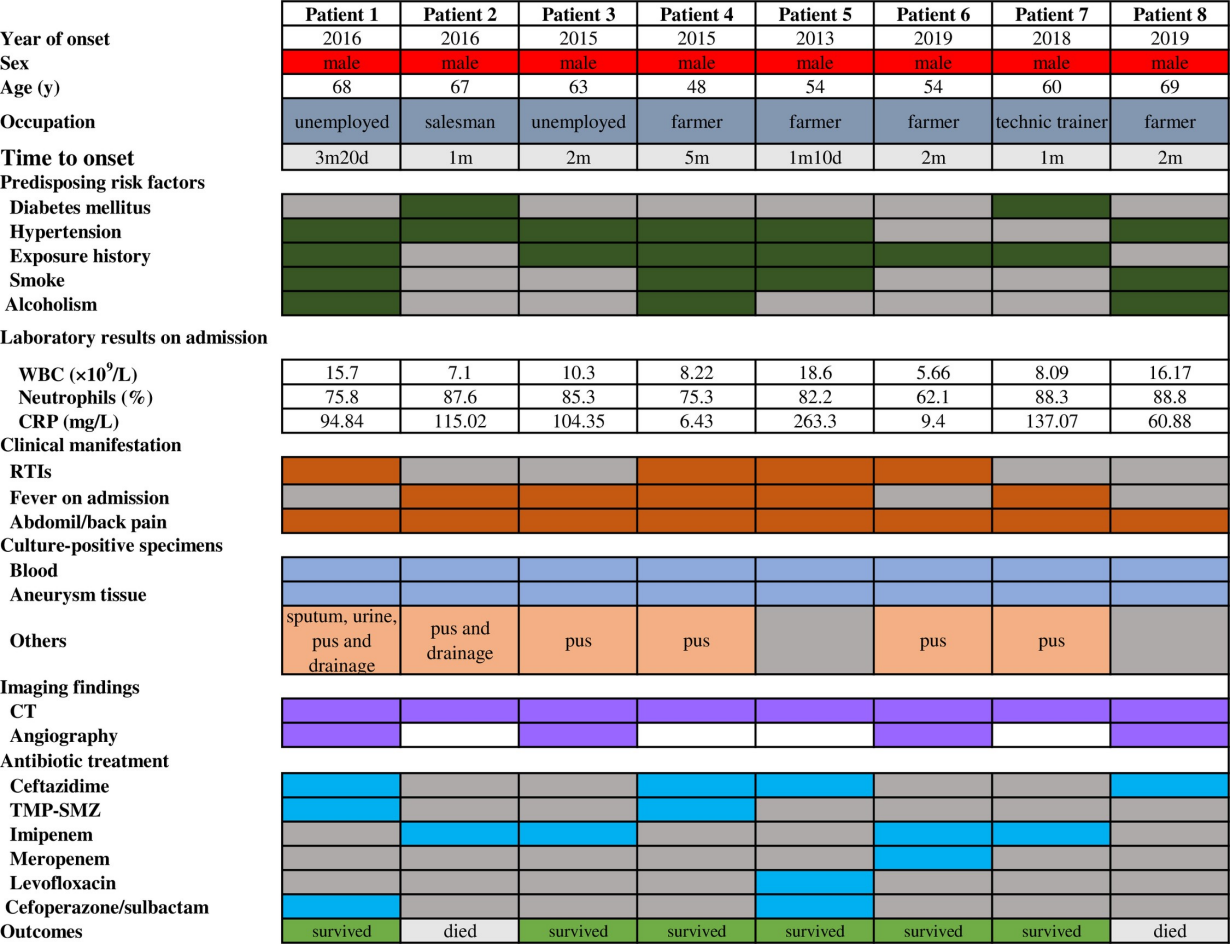
Clinical characteristics and epidemiological features of eight patients with mycotic aneurysms secondary to melioidosis in Hainan province, China: 2013–2019. RTI: Respiratory tract infections; CT: computed tomography; TMP-SMZ: trimethoprim/sulfamethoxazole; WBC: white blood cell; Blank cell: data not available; Grey cell: no; other colored cells: yes. **Notes:** in eight mycotic aneurysms secondary to melioidosis cases, all had positive detections in the CT scan of the abdomen and pelvis. However, by comparison, in 52 of bacteremic melioidosis without mycotic aneurysm, 44 had positive CT results.

In these eight patients, a total of 26 *B*. *pseudomallei* isolates were recovered from bloodstream (8 isolates), aneurysm tissues (8 isolates), pus or drainage (8 isolates from 6 patients), sputum (1 isolate from Patient 1) and urine (1 isolate from Patient 1). All aortic tissues had been sent for pathological examination in the Department of Pathology. Furthermore, we reviewed their medical reports, which included the following variables: demographic features (age, gender and occupation), clinical characteristics (symptoms, mortality, and laboratory and imaging results), potential risk factors (underlying conditions including hypertension, diabetes mellitus, alcoholism, and smoking history), and suspected exposure to water and soil. These were detailed in Table 1, Fig 1, and Fig 2. All these strains were forwarded to the Department of clinical microbiology of Hainan General Hospital. Definitive identification was conducted by the VITEK Compact 2 (BioMerieux, France) card and 16S rRNA gene sequencing.

**Table 1:** Comparisons between the melioidosis patients with mycotic aneurysm and those without.

Characteristics	Total (n = 159)	Mycotic aneurysm secondary to melioidosis (n = 8)	Bacteremic melioidosis without mycotic aneurysm (n = 151)	*x*^*2*^	U	*P*
**Demographic features**						
Median age, y (IQR)	54 (46,60)	61.5 (54,67.8)	54 (46,60)		365.5	0.060
Sex (male), no, %	140, 88.1%	8, 100%	132, 87.4%			0.597
**Clinical Characteristics or underlying diseases**						
Febrile (>38°C) on admission, no, %	110, 69.2%	6, 75.0%	104, 68.9%	0		1
Exposure to soil and dust inhalation, no, %	139, 87.4%	6, 75%	133, 88.1%	0.292		0.589
Diabetes mellitus, no, %	120, 75.5%	2, 25%	118, 78.1%	8.899		0.003
Long-term smoking, no, %	58, 36.5%	4, 50%	54, 35.8%	0.192		0.661
Excessive or long-term drinking, no, %	48, 30.2%	3, 37.5%	45, 29.8%	0.005		0.947
Hypertension, no, %	38, 23.9%	6, 75.0%	32, 21.2%	9.317		0.002
**Death, no, %**	42, 26.4%	2, 25%	40, 26.5%	0		1

Notes*: The CT results were available only in 52 of 159 patients. IQR: interquartile range. CT: Computed Tomography.

### Whole genome sequencing (WGS)

To elucidate the genetic characteristics of the above *B*. *pseudomallei* isolates, we performed whole genome sequencing on each organism collected from blood samples using a whole-genome shotgun sequencing strategy based on the Illumina HiSeq platform.

### Antibiotic susceptibility testing (AST)

The E-test method (Liofilchem, Italy) was used to determine the susceptibility of all the above-mentioned eight isolates to imipenem, ceftazidime, amoxicillin/clavulanate, doxycycline, and trimethoprim/sulfamethoxazole (TMP-SMZ). The results were interpreted in accordance with the breakpoints set for *B*. *pseudomallei* in M45 by the Clinical and Laboratory Standards Institute [16].

### Statistical analysis

We evaluated differences of clinical features and demographics among the groups with and without mycotic aneurysm *via* the Mann-Whitney U test for continuous variables (expressed as the median) and χ2 tests for categorical variables, as appropriate. Statistical analyses and data sorting were conducted using GraphPad Prism version 8.0.1. A *P* value of less than 0.05 was considered statistically significant. Minimum inhibitory concentrations (MIC) data of each antibiotic were recorded and analyzed by WHONET 5.6 software.

## Results

### Demographic features

For 159 patients with culture-confirmed bacteremic melioidosis, the mean age was 53.6±14.0 years, and the median age (interquartile range) was 54(46,60) years, ranging from 2 to 88 years. A total of 25 patients (25/159, 15.7%) were older than 65 years, 41(25.8%)≧60 years and 104 (65.4%)≧50 years. The gender ratio of male/female was approximately 7:1 (139/20) with significant difference. Diabetes mellitus was the most frequent risk factor (120/159, 75.5%). Pneumonia was the most frequent clinical manifestation (58/159, 36.5%), but the patients did show a wide spectrum of clinical features, including soft tissue infection (35/159, 22%), genitourinary infection (29/159, 18.2%), and internal organ abscesses (16/159, 10.1%).

In this retrospective analysis, all 159 patients were evaluated for mycotic aneurysm, of whom 52 cases (32.7%) with underlying risk factors had contrast-enhanced CT scan to evaluate for the presence of an aneurysm, the aneurysm morphology, and the presence of rupture. These potential subjects of mycotic aneurysm included those with fluctuating swelling mass in abdomen, abdominal vascular noise, abdominal pain and fullness, and flank pain [17]. During the study period, all surviving patients were followed up for the occurrence of mycotic aneurysm, but no newly-reported cases were documented. Eight (8/159, 5.0%) suffered from mycotic aneurysms, including six infectious abdominal aortic aneurysms (iAAA) (Patient 1–5, 8, 75%), one (Patient 7) left iliac pseudoaneurysm and one (Patient 6) infectious mesenteric pseudoaneurysm. They are aged 48~69 years, with a median age of 61.5years (interquartile range: 54.0~67.8 years). All were male. All but one (Patient 5) had predisposing risk factors, mainly involving hypertension (6 cases, accounted for 75%), diabetes mellitus (2 cases, 25%) and long-term smoking history (4 cases, 50.0%). Six patients were febrile on admission. The overall case-fatality rate was 25% (2 cases) even after both surgical and antibiotic treatment. At least one abdominal organ supplied by abdominal aorta demonstrated abnormal imaging manifestations. The demographic, epidemiologic, and clinical features of the eight patients with mycotic aneurysm are summarized in Figs 1 and 2. By comparison, among 151 patients without vascular involvement, 78.1% (118/151) had diabetes mellitus and 35.8% (54/151) were long-term smokers. The clinical features and predisposing risk factors between the eight patients with mycotic aneurysm and 151 without were compared and presented in Table 1.

### Antimicrobial susceptibility profile of eight non-repetitive *B*. *pseudomallei* isolates

A total of 26 *B*. *pseudomallei* isolates were collected from specimens in the eight mycotic aneurysm patients secondary to melioidosis. To confirm their identity, 16S rRNA sequencing analysis was performed, and showed that the sequences of the isolates collected from the same patient were identical. The AST was performed for the eight non-repetitive isolates recovered from bloodstream. All were susceptible to ceftazidime and imipenem, and one isolate (Isolate 4) was intermediate to co-amoxiclav with a MIC of 12/6μg/mL and resistant to doxycycline with a MIC of >256μg/mL, as shown in Table 2. The resistance genes of OXA-42, OXA-43, OXA-57, and OXA-59 were identified in all eight isolates using WGS.

**Table 2:** Minimal inhibitory concentration results of the eight non-repetitive *B*. *pseudomallei* isolates.

Antimicrobial susceptibility results (μg/mL)	Breakpoints	MIC of the eight isolates								Range
		Isolate 1	Isolate 2	Isolate 3	Isolate 4	Isolate 5	Isolate 6	Isolate 7	Isolate 8	
Imipenem	S≦4, R≧16	0.50	0.75	0.75	0.75	0.25	0.5	0.5	0.38	0.25~0.75
Ceftazidime	S≦8, R≧32	2	4	2	4	1	3	2	2	1~4
Amoxicillin/clavulanate	S≦8/4, R≧32/16	2/1	4/2	8/4	12/6^I^	2/1	8/4	8/4	8/4	2/1~12/6
Doxycycline	S≦4, R≧16	2	2	6	>256^R^	2	3	12	3	2~125
TMP-SMZ	S≦2/38, R≧4/76	0.25/4.75	2/38	4/76	2/38	0.5/9.5	2/38	2/38	2/38	0.25/4.75~4/76

Notes: MIC, minimum inhibitory concentration; S, susceptible; I, intermediate; R, resistant; MIC range, range of minimum inhibitory concentration; TMP-SMZ: Trimethoprim/sulfamethoxazole

### Genetic profile and AMS phenotype

The genomic sequencing files of the *B*. *pseudomallei* isolates have been deposited at https://submit.ncbi.nlm.nih.gov/subs/wgsundertheGenBank accession no. JAAHUI000000000, JAAHUJ000000000, JAAHUK000000000, JAAHUL000000000, JAAHUM000000000, JAAHUN000000000, JAAHUO000000000, and JAAHUP000000000.

Multilocus sequence typing (MLST) scheme of *B*. *pseudomallei* was conducted by extracting seven housekeeping loci (namely, *ace*, *gltB*, *gmhD*, *lepA*, *lipA*, *nark*, and *ndh*) as described on https://pubmlst.org/bpseudomallei/fromWGS. The sequence types (STs) and allelic numbers were subsequently further identified by querying the MLST database(https://pubmlst.org/bpseudomallei/info/Bpseudomallei_primers.html). A total of four STs were distinguished in eight *B*. *pseudomallei* isolates, represented by ST30 (2 isolates), ST46 (4), ST58 (1), andST1090 (1). All eight STs have been submitted to the above MLST website.

### Literature review

**Fig 3:**
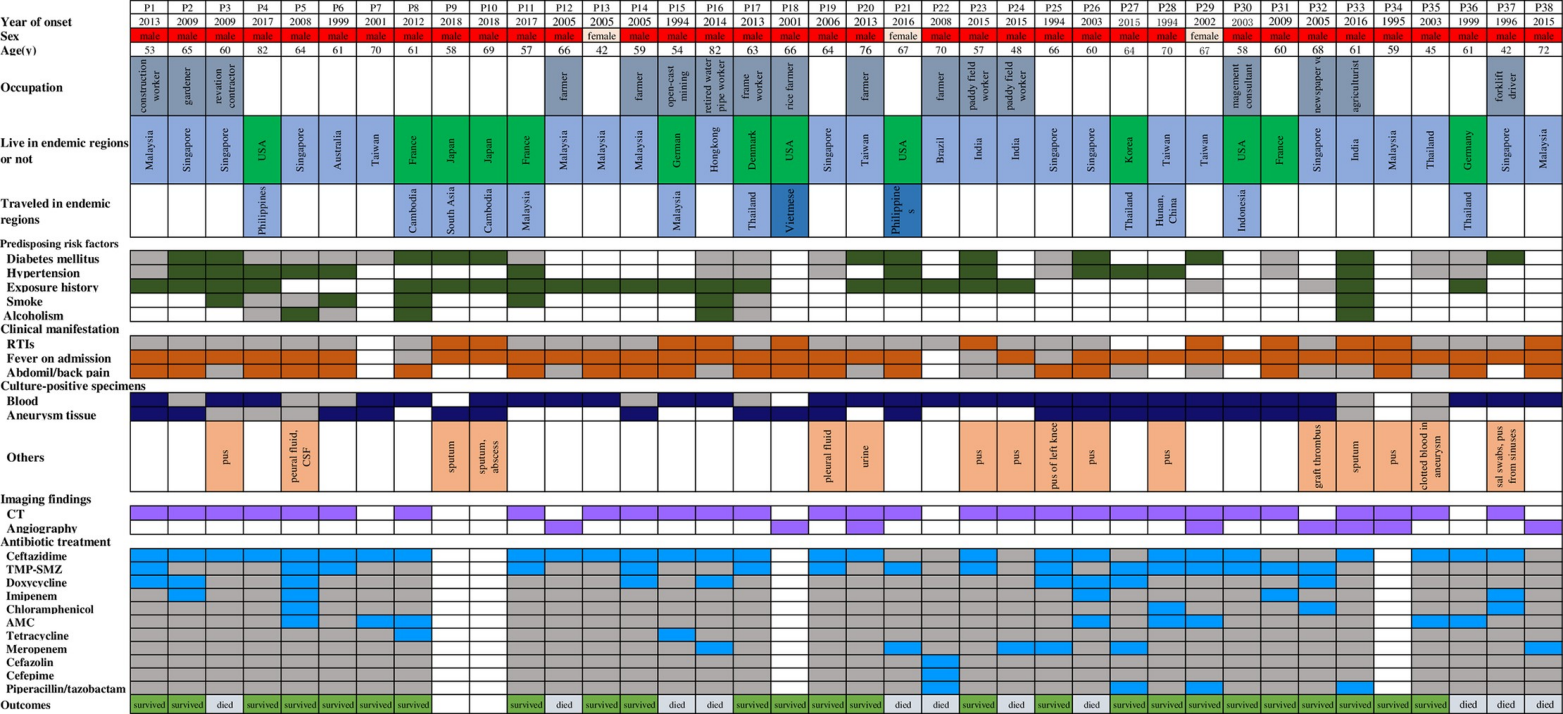
Summary of the 38reported cases of mycotic aneurysm secondary to melioidosis in the literature. RTI: Respiratory tract infections; CT: computed tomography; TMP-SMZ: trimethoprim/sulfamethoxazole; AMC: amoxicillin/clavulanate; Blank cell: data not available; Grey cell: no; other colored cells: yes. Cases, including Case 1 [21], Case 2–3 [4], Case 4 [1],Case 5 [20],Case 6 [22],Case 7 [48],Case 8 [9], Case 9–10 [23], Case 11 [24], Case 12–14 [25], Case 15 [26], Case 16 [3], Case 17 [27], Case 18 [28], Case 19 [29], Case 20 [30], Case 21 [31], Case 22 [19], Case 23–24 [6], and Case 25–26 [10], Case 27 [52], Case 28 [18], Case 29 [32], Case 30 [33], Case 31 [41], Case 32 [35], Case 33 [36], Case 34 [8] Case 35 [37], Case36 [38], Case37 [39], and Case38 [40].

To better understand the features of mycotic aneurysm secondary to melioidosis worldwide, we searched the MEDLINE database (https://www.ncbi.nlm.nih.gov/pubmed) for the studies reporting similar cases. A total of 38 mycotic aneurysm cases caused by *B*. *pseudomallei* were included for comparison, and the details are summarized in Fig 3 [1, 3, 4, 6, 8–10, 18–40].

In the 38 involved cases, 25were inhabitants of endemic areas (Southeast Asia and Australia). In 13 cases living where *B*. *pseudomallei* is considered currently absent, 10 returned from traveling in endemic areas, two were Southeast Asians living in United States, and the data of a 60-year-old African man was unavailable [41]. Furthermore, the subjects involved were aged 42~82 years, with a mean age of 62.3 years, only three were females (3/38, 7.9%). The predisposing risk factors, according to the data available in the literature, mainly involved hypertension (12/20, 60.0%) and diabetes (12/25, 48.0%). In line with data available, 33patientswere febrile on admission, 21cases had possible exposure history, and 28cases had bacteremia. The overall case-fatality rate was 30.6% (11/36, the data of two cases was unavailable).

## Discussion

Mycotic aneurysm is a very infrequent presentation of melioidosis that might be missed [1, 26]. Compared with melioidosis alone, mycotic aneurysm secondary to melioidosis might result in the rupture of the aneurysm, and is associated with high mortality rates [42]. Its diagnosis requires a combination of history findings, clinical features, and radiological and microbiological confirmation [4, 14]. For general population in China, the abdominal aortic aneurysm in at-risk residents is 0.33%, and in 1582 consecutive patients with atherosclerotic risk factors and undergoing coronary angiography, the prevalence of abdominal aortic aneurysm is up to 1.6% in the whole study population and 2.9% in male patients aged over 65 years [43, 44]. Comparatively, the incidence of mycotic aneurysm in the present study was 5.0% (8/159), which might be still under-diagnosed. In addition, it should be noted that 5% represents the proportion of aneurysms in bacteremic melioidosis cases and the overall proportion for all melioidosis cases should be around half that. The increasing articles in the literature have reported the epidemiological data of mycotic aneurysm secondary to melioidosis in various regions [1, 3, 6, 12]. Our literature review showed that the incidence of mycotic aneurysm caused by *B*. *pseudomallei* was geographically varied, for example, 0.4% (2 of 540 cases of melioidosis) in Australia [13] and 7.5% in Malaysia (5 of 67 cases of melioidosis 1975 to 2015) [11, 45]. The low proportion of cases in Australia compared with Malaysia might be also explained by a higher proportion of low-income households and less access to medical resources in Southeast Asian countries [11, 13, 45]. Interestingly, in accordance to the literature review revealed in Fig 3, the preponderance of cases was amongst the developed country travelers from developing countries, which also suggests that mycotic aneurysm secondary to melioidosis is actually being missed in endemic areas and diagnosed only when patients are managed in countries that are better resourced and where imaging is more widely available [2, 9, 23, 24, 27, 38].The literature review demonstrated that Hainan was the main epidemic focus of melioidosis in China. In 2011 the Institute for Communicable Disease Control and Prevention of China established a working group to monitor the epidemiology of melioidosis throughout China, revealing the overwhelming majority of culture-confirmed cases were from Hainan (99.0%, 392/396, 3 from Guangxi and 1 from Guangdong) between 2002 and 2016, and septicaemia was the most common clinical manifestation (153/277, 55.2%) [15].Taken together, the mycotic aneurysm cases collected in Hainan Province in the present study can represent the general epidemiological features in China.

It is documented that most patients with infected mycotic aneurysm secondary to melioidosis are male and aged 60 years [1, 2, 12, 19]. Similarly, in our case series, the ages of eight subjects ranged from 48 to 69, with a median age of 61.5 years, all were indigenous males, and two died at the age of 67 and 69, respectively. That there is a considerable preponderance of males might be explained by the fact that males are more likely to come into contact with contaminated soil and water while working outdoors. For example, 92% (105/114) melioidosis were males in southern India [46], and59% (1314/2243) in Thailand [47]. In the current study, four patients were field workers and probably got this infectious agent due to high environmental exposure. This is in keeping with other literature reviewed, as shown in Fig 3 [1, 3, 4, 6, 9, 10, 19–31, 48], namely, in 38 cases of mycotic aneurysms secondary to melioidosis, 92.1% (35/38) were male and 68.4% (26/38) were aged over 60 years with an average age of 62.3 years. Interestingly,73% (56/77) imported melioidosis cases into Europe were also males, with the sex ratio of male/female at 2.7 [49].All they were returned travelers, with a mortality rate of6% [49]. It maybe that females are more risk averse and hence reduced exposure. Further studies should be conducted to elucidate the male predominance.

The abdominal aorta is mostly detected in an infected mycotic aneurysm [4, 6], however, thoracic mycotic aneurysm, coronary aneurysm, iliac aneurysm, intracranial mycotic aneurysm, and renal artery pseudoaneurysm might also be involved [6, 8, 32, 39, 50, 51]. In our study, six iAAAs were identified, but infectious mesenteric pseudoaneurysm (1 case) and left iliac artery pseudoaneurysm (1 case) were also observed.

Furthermore, the patients with mycotic aneurysm secondary to melioidosis often have predisposing risk factors, including smokers, alcoholism, diabetes and exposure to contaminated soil or water prior to their illness [4, 14]. In line with our literature review, there were 10 cases (10/38, 26.3%) returned from endemic regions. Taken together, if the suspicious cases of infected mycotic aneurysm occurred in non-endemic regions, the clinicians should be alert to their travelling history [9, 27, 33, 52]. Moreover, increased awareness of the risk factors of aneurysms of the aorta is also urgently required. In a previous study of 40 infected mycotic aneurysms (80% male, and mean age 63 years), *B*. *pseudomallei* was the most common pathogen recovered (17/40, 42.5%), and diabetes and hypertension were the most common comorbid conditions [7, 12]. In accordance with the data available in the literature, hypertension (12/19, 60%) and diabetes (12/25, 48.0%) were closely related to mycotic aneurysm secondary to melioidosis. This is true in the present case series, too: all six iAAA patients have hypertension and two have diabetes mellitus. The risk factors that predispose to developing aneurysms, hypertension and diabetes, are present in cases of mycotic aneurysms is no surprise as these are likely present prior to infection which become secondarily infected, with a minority being primary. However, it is difficult to prove and further studies are required.

However, the two subjects with infectious mesenteric pseudoaneurysm and left iliac artery pseudoaneurysm had no hypertension. All eight patients had lived and worked in Hainan province, endemic to melioidosis in China; four (4/8, 50%) presented with pneumonia and only two (2/8, 25.0%) had diabetes. Melioidosis might manifest as a pulmonary infection that mimics pulmonary tuberculosis and tended to be misdiagnosed [2, 53]. In our report, Patient 4 had a coinfection of pulmonary tuberculosis and melioidosis. Furthermore, a previous study demonstrated that post-traumatic infectious pseudoaneurysm is the main type of infectious aneurysm [42]. Both the Patient 2 and 7 had a history of motorcycle accident, and got an abrasion on their legs and exposed to soil, and later, abscesses formed and they had chill and fever. Atherosclerosis is an independent risk factor for the development of abdominal mycotic aneurysms suggested by Toghill et al. [54]. Four of the eight patients (Patients 1, 3, 6, and 8) in this study underwent vascular ultrasonography, and atherosclerosis or plaque was seen in their arteries.

Understanding of clinical features of the infectious aneurysm by *B*. *pseudomallei* should be made for reducing the mortality rate. Most patients with infectious aneurysms were febrile [1, 12, 22]. In line with the literature review, 33 patients (33/38, 86.8%) were febrile on admission. However, some subjects were afebrile [6, 9, 10]. In a Thailand study, only 76.5% of the patients with mycotic aneurysm were febrile [12]. In the present study, three out of eight mycotic aneurysm patients were afebrile. All eight patients in this study showed abdominal pain, but local pain might not always be the characteristic feature of the mycotic aneurysm [29, 32, 33]. More attention still should be paid to those with *B*. *pseudomallei* infection and predisposing factors, and then further evaluation should be underscored. Furthermore, as shown in Fig 2, there were 4 cases of leukocytosis (50%) and 6 cases with a high C-reactive protein (75%), hinting that inflammatory biomarkers might play a limited role [17].

Because of the high risk of rupture and susceptibility to recrudescence, urgent in-situ or extra-anatomical repair combined with debridement and appropriate antibiotic therapy is mandatory for an aneurysm [41]. In the present study, six patients were managed with resection of mycotic aneurysm and reconstruction followed by medical treatment and were discharged uneventfully. Two patients died of mycotic aneurysm rupture, infectious shock, and abdominal mycotic dissection rupture and bleeding, before the infecting etiological agent was identified, similar toa previous study [6]. Therefore, Laboratory diagnosis is still made by conventional blood and mycotic tissue culture techniques; the rapid and early recovery of the principal etiological agent, if the infected aneurysm was under suspicion, will be helpful for the possible recovery of the patients [6].

Presently, positive blood culture together with a radiological abnormality of the artery in the abdomen and pelvis (mainly on CT imaging) strongly suggests an infected mycotic aneurysm [19]. Herein, in our cases, at least one organ imaging abnormality was present in the intra-abdominal organs supplied by the abdominal aorta. All eight patients were identified to have mycotic aneurysm through CT, and all cultures of blood and artery tissue specimens grew *B*. *pseudomallei*. Therefore, we suggest that, for patients who present with positive blood culture for *B*. *pseudomallei* and abdominal pain, early CT scan of the abdomen and pelvis should be considered for timely diagnosis and intervention, as this may lead to a reduction in morbidity and mortality [41].

There are limited antibiotics for *B*. *pseudomallei*. Ceftazidime, imipenem or meropenem, amoxicillin/clavulanic acid, and oral TMP-SMZ, are the mainstay in the treatment of infections by *B*. *pseudomallei* [1, 4, 6, 9, 10, 20–33, 48, 52]. According to the present study and documents reviewed in Fig 3, ceftazidime and TMP-SMZ were the top two antibiotics used in the treatment of *B*. *pseudomallei* bacteremia. Our eight *B*. *pseudomallei* isolates remained high susceptibility rates to the above antibiotics. However, the *B*. *pseudomallei* isolate of the patient 4 was insusceptible to amoxicillin/clavulanic acid and doxycycline. Fortunately, he was administrated with ceftazidime and TMP-SMZ. Moreover, there is no relapse in our mycotic aneurysm cases; relapse is one of the most important complications of melioidosis, with a recurrent rate ranging from 6% to 23% [47, 55, 56]. Although most of the strains were susceptible to antimicrobials, inadequate source control [57], choice of antimicrobial [58], nonadherence or duration less than 12 weeks [58, 59] are among the most important determinants of relapse.

Moreover, ST 30 (2 isolates), ST46 (4), ST58 (1), and ST1090 (1) were identified in our eight *B*. *pseudomallei* isolates, different from previous reports [5, 60]. Except for ST1090, the other three STs have been reported in the *B*. *pseudomallei* strains causing bacteremia in China (https://pubmlst.org/bpseudomallei/).

Our study is obviously limited by several factors. First, the sample size is relatively small within the study time period, even though the small sample size is not uncommon in this type of study, therefore reducing the robustness of the clinical characteristics of the mycotic aneurysm caused by *B*. *pseudomallei*. The conclusions should be interpreted with caution, thus further studies are still needed to provide further insights into our knowledge. Second, the major limitation of the study is the retrospective study design, which might result in selection bias.

In summary, our report will allow for an in-depth understanding of mycotic aneurysm secondary to melioidosis in China. The mycotic aneurysm is a recognized manifestation and complication of melioidosis with high mortality if not managed appropriately or in a timely manner. Furthermore, it may not be rare, being far commoner than previously realized, up to 5.0% of bacteremic cases in our series. Many protean manifestations of the disease have been demonstrated and high clinical vigilance is required from clinicians and other specialties in endemic regions. The early diagnosis through imaging should be under consideration in febrile males with abdominal pain and hypertension in regions of melioidosis endemicity, especially when the organism was isolated in blood culture [9, 17]. Early use of appropriate antibiotics and adequate surgical debridement will improve the outcome of patients.
